# A practical online tool to estimate antiretroviral coverage for HIV infected and susceptible populations needed to reduce local HIV epidemics

**DOI:** 10.1038/srep28707

**Published:** 2016-06-24

**Authors:** Antoine Chaillon, Martin Hoenigl, Sanjay R. Mehta, Nadir Weibel, Susan J. Little, Davey M. Smith

**Affiliations:** 1 Division of Infectious Diseases, University of California, San Diego, La Jolla, California, United States of America; 2 Section of Infectious Diseases and Tropical Medicine, Department of Internal Medicine, Medical University of Graz, Graz, Austria; 3 Division of Pulmonology, Department of Internal Medicine, Medical University of Graz, Graz, Austria; 4 Veterans Affairs San Diego Healthcare System, San Diego, California, United States of America; 5 Department of Computer Science and Engineering, University of California San Diego, California, United States

## Abstract

It remains unclear what proportions of HIV-infected and uninfected people should receive effective antiretroviral therapy (ART) to control local HIV epidemics. We developed a flexible model to evaluate the impact of treatment as prevention (TasP) and pre-exposure prophylaxis (PrEP) on HIV incidence in local communities. We evaluated this tool for determining what TasP and PrEP targets are needed to substantially reduce the HIV epidemic in San Diego, which is predominately comprised of men who have sex with men. By increasing the proportion of HIV-infected individuals on ART from 30% to 50%, 686 new infections would be prevented over five years in San Diego. By providing PrEP to 30% of MSM to the age group that account for 90% of local HIV incident cases (21–52 years), we could prevent 433 infections over five years. When combining these initiatives, a PrEP coverage rate of 40% and TasP coverage rate of 34% would be expected to decrease the number of new infections by over half in one year. This online tool is designed to help local public health planners and policy makers to estimate program outcomes and costs that may lead to better control of their local HIV epidemics.

It is becoming increasingly clear that we may now have the tools to end the HIV epidemic, especially with broader use of antiretroviral therapy (ART)^1^. Beyond improving the lifespan and well-being of HIV-infected individuals, ART can also be used as (1) treatment as prevention (TasP)^2^, (2) pre-exposure prophylaxis (PrEP)^3^, (3) post-exposure prophylaxis and (4) microbicide. The effectiveness of ART to reduce HIV incidence in a population, however, is limited by its uptake among those who are infected (i.e. TasP) and those who are susceptible (i.e. PrEP, PEP, and microbicides). This is especially poignant since only about 25% of HIV-infected people in the United States^4^,^5^ and 30% in San Diego^6^ now are diagnosed and on suppressive ART, and the number of highly susceptible HIV-uninfected people on PrEP is considerably lower^7^. The proportions of HIV-infected individuals on effective ART and susceptible HIV-uninfected individuals on PrEP that are needed to control an epidemic in a population remain unknown.

Empirical and deterministic models have been developed by several groups to evaluate the impact of voluntary HIV testing and immediate ART^8^, the impact on HIV prevalence and incidence of interventions that decrease disparities^9^ or cost-effectiveness of treatment and prevention^10^,^11^. While these studies are highly relevant to determine the theoretical impact of initiatives, they are complex and less conducive for adjustments for local HIV epidemics, especially when quickly comparing the adjustment of epidemic or initiative parameters to suit local efforts. In this setting, simple mathematical models using empirical parameters, as provided here, may help provide targets of TasP and PrEP coverage rates to reduce HIV incidence in local populations.

Focusing on a community-based response, we developed an intuitive and flexible tool to estimate the potential impact of TasP and PrEP coverage rates on HIV incidence in a population. We evaluated our tool on the HIV epidemic in San Diego, using previously measured local HIV epidemic parameters^12^; however, the tool can be adapted to any population that has accurate estimates of epidemic parameters. We believe that such a tool may help to provide estimates of what proportion of HIV-infected and uninfected people in a local population need to be covered with ART (TasP and PrEP) to most efficiently control an epidemic.

## Methods

This study aimed to create a practical tool to estimate the potential impact and costs of combined PrEP and TasP strategies on HIV incidence in a local population. We then evaluated this tool in the setting of the HIV epidemic in San Diego. To provide maximal flexibility and utility, we limited the number of input variables and proposed default parameters based on published data. Since local conditions can influence each of these variables, we designed our tool (http://gtzero.ucsd.edu/) to allow parameters to be adjusted.

### Model Description

#### Number of new HIV infections

We used a standard crude rate of growth model to derive the number of new HIV infections over time in a given population. The population of viremic HIV-infected individuals was defined from the initial population size (P_0_) based on a TasP coverage rate (ƒ) with an average TasP efficacy of 80%^13^ for a HIV prevalence (*π*). Hence:


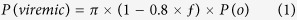


The population of susceptible individuals *P(susceptible)* was estimated based on initial population size *P(o)* and the HIV prevalence (*π*). Assuming a PrEP coverage rate (**∂**) with an average PrEP efficacy of 70%^3^, *P(susceptible)* would be:


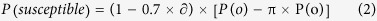


Considering results of previous studies to estimate the HIV transmission risk per sex act (ß) for condomless receptive anal intercourse (CRAI) and condomless insertive anal intercourse (CIAI)^14^, we first estimated the overall transmission risk per condomless sexual act as:


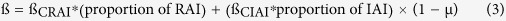


where μ = the frequency of condom use.

Here, the proportion of insertive and receptive sex acts indicate the probability that, for a given sex act between an HIV-positive partner and an HIV-negative partner, the HIV-negative partner is the receptive partner (or the insertive partner).

Then calculated the probability of transmission over (C) acts using the standard binomial formula to estimate the cumulative risk over c exposures ß(c)^15^:


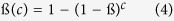


#### Crude Cost Estimates

Next, we incorporated costs into our calculations in relation to infections averted. Since prices change, the provided tool allows TasP and PrEP costs to be adjusted. Assuming that cost estimates for TasP was $24,000 (in 2016 US dollars) from^16^ and for PrEP was $10,300/year from^17^, which included costs of recommended drugs (combination oral emtricitabine/tenofovir disoproxil fumarate) and clinical monitoring, considering U.S. inflation from 2010 to 2016 and fluctuation of costs over time due to multiple other factors among which manufacturer costs, third party payor negotiations, generic pricing, etc we derived the annual gross costs of TasP and PrEP as:


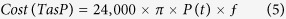



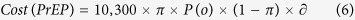


Hence, the overall gross cost of combined TasP and PrEP initiative can be estimated as the sum of (1) and (2).

We also took into account the annual costs saved by new infection averted (NIA) as:


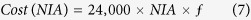


Derived from (1) to (3), the annual net cost for a given TasP coverage rate (ƒ) and PrEP coverage rate (∂) was defined as:


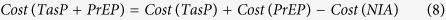


### San Diego HIV Epidemic

We evaluated our tool for the HIV epidemic in San Diego using latest available data (Table 1)^18^. Since the San Diego epidemic is largely centered among MSM (>80%), we focused our model on the MSM epidemic in San Diego, considering a total of 56,000 MSM individuals (*P*_*0*_) and an HIV prevalence (*π)* of 20%^19^.

#### Coverage rate of effective ART among HIV-infected individuals

Currently, around 30% of persons living with HIV infection in the United States are prescribed ART and achieve viral suppression^13^, which is similar to data for San Diego^18^.

#### Efficacy of TasP

Use of ART can reduce HIV transmission by 92–98%^2^,^20^. Other studies have less optimistic estimates^21^, so we set the efficacy of TasP at 80%, but have also included a sensitivity analysis for this parameter.

#### Efficacy of PrEP

Based on the iPrEX study^3^, the efficacy of PrEP in men who, by self-report and pill count, took the drugs more than 90% of the time was 73%, so we conservatively estimated an average of 70% efficacy of PrEP to prevent HIV infection in among MSM in San Diego. Of note, the overall efficacy of belonging to the active PrEP arm in the iPrEX study was 44% and local conditions are likely to vary; therefore, the model allows this parameter to be modified directly by the user.

#### HIV transmission risk per sex act (β)

The risk of HIV per sexual act varies among risk groups. For San Diego, we used estimates from a recent review where MSM risk was estimated at 138 infections per 10,000 exposures for CRAI and 11 per 10,000 exposures for CIAI^14^. For the following population-based analyses, we considered an equal proportion of insertive and receptive acts (1:1).

#### Number of sex acts with casual partners per year (*C*)

Since the San Diego HIV epidemic is predominately a sexual risk epidemic among MSM and we did not have an empiric measurement of the average number of sexual contacts with casual partners (*C)* among MSM in San Diego, we stratified our analyses by the mean number of sex acts with a casual partner (from 10 to 30 per year), which encompasses previous estimates provided by an Australian study that followed 1,427 MSM over 6 years^22^.

#### Condom use

Since condom use reduces sexual risk, we included estimates of condom use based on data from San Diego MSM reported in 4 categories: i) ‘never’ (0%), ii) ‘sometimes’ (1–50%), iii) ‘mostly’ (51–99%) and iv) ‘always’ (100%)^12^ (Fig. S1). From these data, we estimated an average condom use of 60% for anal sex.

#### Age targeted PrEP

Targeting PrEP to those at highest risk would likely be more efficient^23^. To evaluate if PrEP can be targeted to reduce HIV incidence while reducing costs, we estimated: (i) age distribution of men in San Diego from^24^, (ii) number of MSM in San Diego (*P*_*0*_) from, and^18^ (iii) young key MSM population in San Diego as the age group who account for 90% of new HIV infections from^12^ Hence, MSM in San Diego aged between 21 and 52 years old accounted for 46.3% of all MSM^24^ but represented 90% of all new HIV infection (Fig. S2).

Since 10% of at risk MSM will not have received any PrEP (∂ = 0) as they do not fulfill criteria established for the 90% at highest risk, we defined the number of new HIV infections with PrEP targeted by age as:


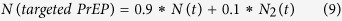


where *N*_*1*_*(t)* is the number of new HIV infections for a given PrEP coverage rate (∂) and *N*_*2*_*(t)* is the number of new HIV infections without PrEP (∂ = 0).

We also defined the cost estimates for PrEP targeted based on age as:


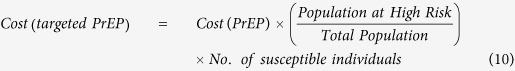


Hence in San Diego:


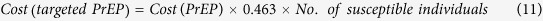


### Sensitivity Analyses

To evaluate the impact on HIV incidence and costs of combined TasP and PrEP with and without PrEP targeted on age, we performed sensitivity analysis on: i) non-targeted PrEP coverage versus TasP coverage rates, and ii) age targeted PrEP coverage versus TasP coverage rates. We described the number of new HIV infection after one and five years and the cost per NIA for combined PrEP and TasP prevention strategies 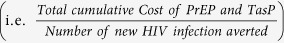. We also evaluated the potential impact of sexual risk compensation on the number of new HIV infection via sensitivity analysis based on PrEP coverage rate (∂), condom use frequency, and the average number of sexual acts with casual partner per year (*C*).

### Role of the funding source

The funding source had no role in the design and conduct of the study; collection, management, analysis, and interpretation of the data.

## Results

It has been touted that local HIV epidemics can be controlled with TasP and PrEP)^3^, but it is not clear what rates of TasP and PrEP are needed. To this end, we created and evaluated a tool that can provide estimates of TasP and PrEP coverage rates needed to reduce HIV incidence. We present this tool utilizing parameters for the HIV epidemic in San Diego, California, but the flexibility of the tool should allow for any local epidemic driven primarily by sexual risk to be evaluated.

### HIV in San Diego

Around 10,710 HIV-infected individuals live in San Diego, California^18^. The vast majority of these individuals are MSM (82%)^18^, and around 30% are receiving ART with suppressed viral loads^13^, while the proportion of susceptible MSM receiving PrEP is likely negligible^25^. Based on a mean of 20 sex acts per year, the cumulative number of newly infected individuals in San Diego would be expected to grow by 449 in one year and 2,291 in five years (Table S1). These numbers are congruent with local estimates reported by the Health and Human Services Agency of San Diego County in ref. 18.

### Impact of TasP

**Figure 1:**
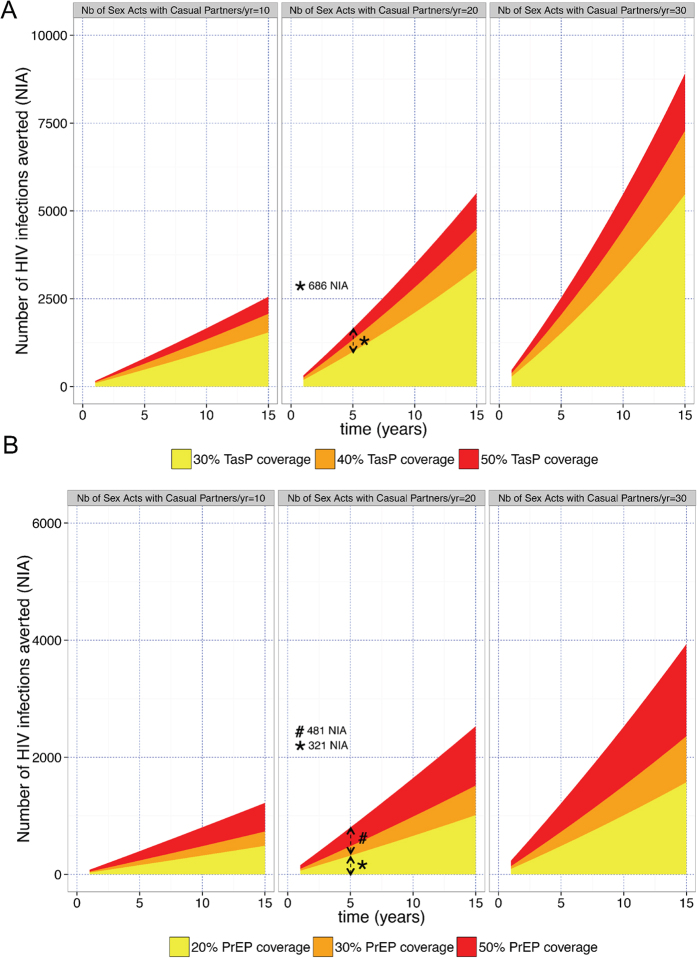
TasP coverage rate (**A**) and PrEP coverage rate (**B**) and estimated number of new HIV infections averted (NIA) among MSM in San Diego. The number of HIV infections averted (NIA) is proposed for: (**A**). Three different levels of TasP coverage rate are presented in yellow (30%), orange (40%) and red (50%). (**B**) Three different levels of PrEP coverage rate are presented in yellow (30%), orange (40%) and red (50%). These analyses were based on an initial population size of 56,000 MSM, an HIV prevalence of 20% among MSM, a mean number of sex acts of 10, 20 and 30/year, and 60% condom use, and are stratified by three levels of number of sex acts with casual partner (10, 20 and 30/year).

In San Diego, if TasP coverage rate increased from the current rate of 30% to 40% coverage (Table S1), then 344 new infections could be averted over 5 years, and at 50% TasP coverage rate 686 new infections could be averted (Fig. 1A, see*, Table S1). Gross cost estimates of increasing TasP from 30% to 50% coverage would be $286 million over 5 years (Fig. S3A, Table S2). Interestingly, if TasP coverage rate was 50%, then gross annual costs of TasP would be estimated to decrease after 10 years (Fig. S4).

### Impact of PrEP

Considering a scenario of 30% TasP coverage rate and an average of 20 sex acts per year, increasing PrEP coverage rate to 20% of all MSM in San Diego would prevent 321 new HIV infections after 5 years compared to a scenario without PrEP (Fig. 1B, see*, Table S1). Increasing PrEP coverage rate from 20% to 50% all MSM in San Diego would avert 481 more infections (i.e. 1810 NIA compared to no PrEP, Fig. 1B, see^#^, Table S1). The cost of covering 30% of all MSM in San Diego would cost $584 million over 5 years (Fig. S5, see*, Table S2). Unlike for TasP, the costs of PrEP did not decrease with higher rates of PrEP coverage rate or over time.

### PrEP Targeted by Age

**Figure 2:**
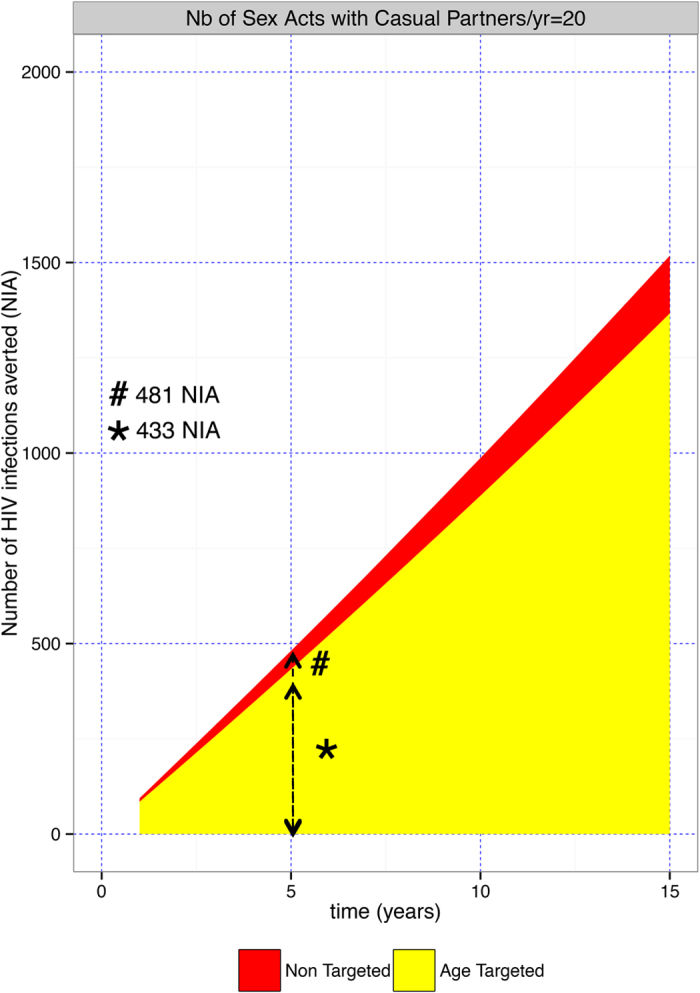
Number of new HIV infections averted (A) by targeting PrEP to MSM between the ages of 21 and 52 years. The number of new HIV infections averted are estimated for a PrEP coverage of 20% with an average number of sex acts with causal partner is 20/year.

While MSM are the main risk group in San Diego, the risk of infection is not uniform^26^. We estimated that 90% of all new HIV infection among MSM in San Diego occurred among men aged between 21 and 52 years old^12^. This young key population accounted for 46.3% of all MSM in San Diego^24^. If PrEP was targeted to 30% of MSM between the ages of 21 and 52 years, it would avert 433 infections over 5 years (Fig. 2, see*), costing around $270 million (Fig. S5, see*, and Table S3 in the Supplement, shaded box). By comparison, if PrEP was provided to 30% of all MSM it would avert 481 new infections over 5 years (Fig. 2, see^#^, Table S2), costing around $580 million dollars (Fig. S5, see^#^).

**Figure 3:**
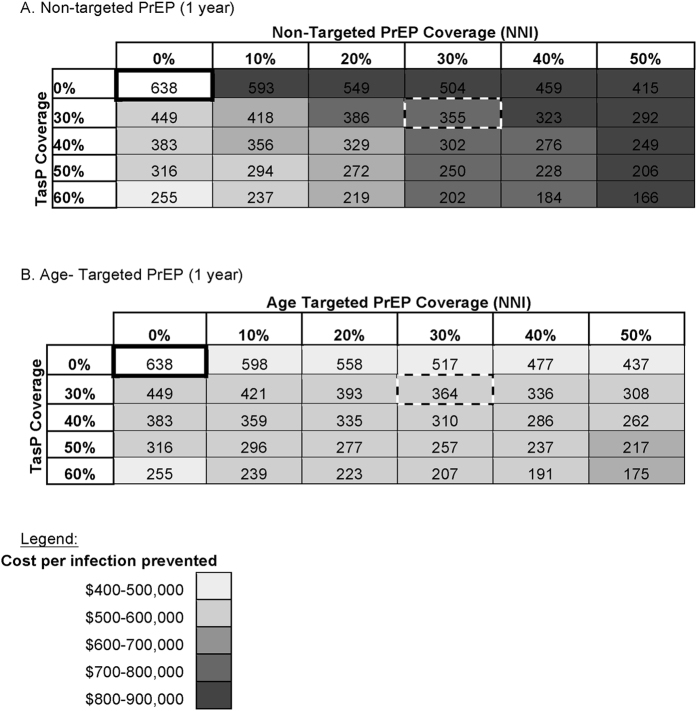
Number of new infections (NNI) and cost (shading) of Non-targeted (**A**) and Age Targeted (**B**) PrEP and TasP coverage rate in San Diego. The NNI is showed after 1 and 5 years for each scenario. Boxes are colored according to the cost in million USD per infection averted compared to a scenario without PrEP and TasP (white bold boxes).

**Figure 4:**
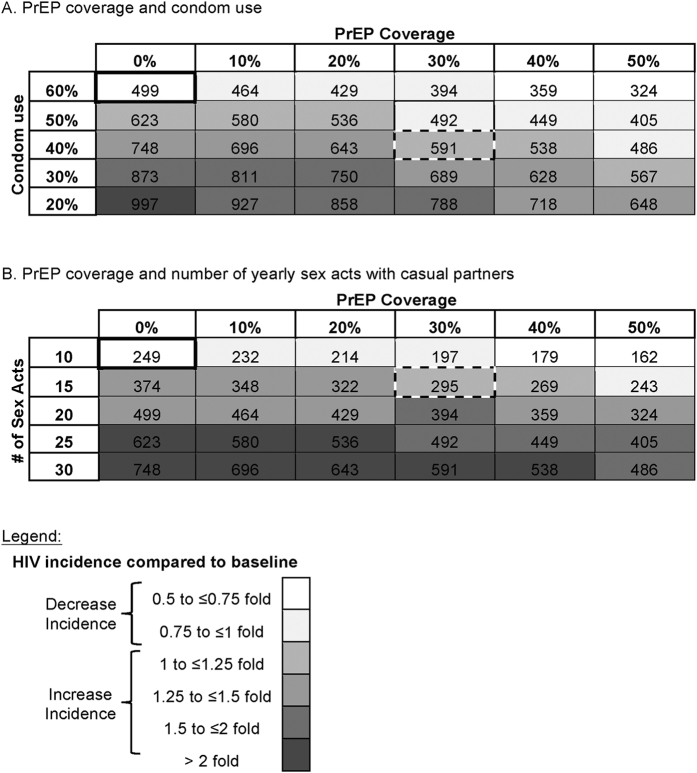
Number of new HIV infections (NNI) in relation to PrEP coverage rate and condom use (**A**) and average number of sex acts with casual partner (**B**). In both scenarios, the number of new HIV infections are provided after one year considering 30% TasP coverage rate. Boxes are colored in grey scale in relation to the baseline NNI when PrEP coverage rate is 0% (white bold box), and darker colors represent change in NNI over baseline. Sexual risk compensation overcame the benefits of PrEP when the NNI is expected to be higher than the baseline NNI when PrEP was 0%.

Since, a variety of combinations of TasP and PrEP coverage rates can reduce HIV incidence among MSM in San Diego, we performed a sensitivity analysis for age targeted versus non-targeted PrEP (Fig. 3). When TasP coverage rate was at 30% of all HIV-infected individuals and PrEP coverage rate at 30% of all susceptible MSM (i.e. non-targeted PrEP), we estimated that this would decrease the number of new infections from 638 to 355 after one year (Fig. 3A). This is similar to when TasP coverage rate remains at 30% and PrEP covers 30% of MSM between the ages of 21 and 52 years (i.e. age targeted PrEP), with number of new infections decreasing from 638 to 364 (Fig. 3B). However, the gross cost per infection averted was less for age targeted PrEP than non-targeted PrEP ($558,000 vs. $767,000, Fig. 4).

### Sexual Risk Compensation

To evaluate the potential impact of sexual risk compensation, we performed a two-way sensitivity analysis based on: (i) PrEP coverage rate, (ii) condom use, and (iii) average number of sexual acts with casual partner per year. First, if overall condom use decreased from 60% to 40% but PrEP coverage rate to all MSM was increased from 0% to 30%, then in one year, the number of new HIV infections would increase from 499 to 591 (versus 394 if condom use had stayed at 60%) (Fig. 4A). Similar to condom use, in one year the number of new HIV infections increased from 249 to 295 when sex acts with casual partners per year increased from 10 to 15 despite a PrEP coverage rate increase from 0% of 30% (Fig. 4B).

### Combined TasP and PrEP

We then evaluated logical benchmarks to better understand what targets of PrEP and TasP coverage rates would be needed to make meaningful impacts on the San Diego epidemic. To reduce the number of HIV infections in San Diego by over half in only one year (638 vs. 317 new infections), simple calculations suggested that it would take an age targeted PrEP coverage rate of 40% and TasP coverage rate of 34%, costing around $183 million. To reduce the number of new infections in San Diego by two-thirds in 20 years would require at least half of MSM aged between 21 and 52 years to be covered by PrEP (age-targeted PrEP coverage rate) and 60% of HIV-individuals receiving effective ART (TasP coverage rate), costing around $4.6 billion. In general, to prevent one HIV infection over one year, we estimated that 17 HIV-infected individuals should be treated with ART or 100 susceptible MSM should receive PrEP. All of these estimates depend on stable rates of sexual risk.

## Discussion

This study developed an intuitive and flexible tool based on the main drivers of sexual HIV epidemics to provide targets of TasP and PrEP coverage rates needed to reduce HIV incidence. By modelling antiretroviral therapy benefits and gross direct costs, this model can also be used to maximizes the returns on investment in the HIV response as defined by the UNAIDS^27^. We evaluated this tool in the context of the well-characterized HIV epidemic in San Diego, which is predominantly driven by risk among MSM, similar to many metropolitan areas across the U.S.^28^. We have also provided this tool in a form that can be adapted for other communities.

In general, all HIV-infected individuals should receive ART as soon as possible for their own health, but this is clearly not occurring in the U.S. and in San Diego, where only about 25% and 30% of HIV-infected individuals are receiving effective ART^6^, Increasing these rates would also decrease ‘community viral load’^29^ and thus overall transmission, i.e. TasP^2^. Similarly, PrEP use among young key populations is also low^7^. Using the developed tool, we estimated what targets of TasP and PrEP coverage rates would be needed to impact HIV incidence. As an example, this tool estimated that it would require 60% TasP coverage rate and 50% PrEP coverage rate of MSM between the ages of 21 and 52 years to reduce the number of new infections in San Diego by 75% in 20 years. However, sexual risk compensation can jeopardize the benefits of prevention initiatives^30^, and we found the potential reduction in HIV incidence by PrEP and TasP coverage rates can be theoretically offset by increased risk behavior. Further, the provided assessment of combined TaSP and PrEP estimated the efficacy of PrEP as 70% among those individuals who adhere to prescribed to PrEP. We feel that this level of efficacy is conservative, given that the iPrEx trial demonstrated a much higher efficacy of prevention among those who regularly took the medication. However, this estimate may also be overly optimistic given that in the blinded randomized control iPrEx trial, a considerable number of participants did not adhere to their medication and thus the overall additional protection of PrEP was only 44%. To this end, we have allowed the provided tool to be adjusted based on local estimates of PrEP efficacy. Similar to estimates on effectiveness to PrEP, our presented estimates for the effectiveness of condom use are overly optimistic and were presented as 100% effective, and perhaps more realistic at 80% effective, as condom use errors may be relatively frequent. Therefore, similar to PrEP effectiveness, we have allowed parameter to be adjusted in the tool.

The provided tool also offers a customizable simple costs calculator that can provide gross estimates of the cost of PrEP and TasP initiatives. It has been previously estimated that a prevention initiative would be cost saving if it cost less than $326,500 per infection averted^31^. In the short term, most of our scenarios for TasP and PrEP had costs that were higher than this, but over time these costs, especially for TasP, decreased as more infections were averted (Fig. S4). As might be expected, we did find that targeting PrEP to MSM between the ages of 21 and 52 years could reduce the cost per new infection averted by 27% for a scenario with 30% TasP and 30% PrEP coverage rates, although always remaining above $326,500 per infection averted. We acknowledge that targeting PrEP to other groups, like very young MSM and minorities, may be even more effective^12^,^32^. These cost estimates are not exact, but we believe that this, perhaps overly simplified, tool can still provide meaningful costs estimates that can at least start the conversation around planning prevention initiatives. The strategic use of antiretroviral medications is important in an optimized effective investment approach in the HIV response^27^. Here, we provide a simple tool for practical planning of Public Health strategies allocating resources towards combinations of TasP and PrEP interventions. This tool is designed to provide an effective investment approach to maximize the returns on investment in the HIV response and enhance impact by tailoring efforts on key locations and populations, as appropriate.

The main limitation of this study is that the model is wrong. As Drs. Box and Draper stated in their book^33^, “Essentially, all models are *wrong*, but some are *useful*.” While perhaps crude, our model used a flexible and simple structure to facilitate translation to other communities for accessible targets for TasP and PrEP coverage rates. An important part of HIV epidemics that this model ignores is the fact that the structure that underlies the transmission network is important^34^. As such, targeted prevention efforts, based on the structure of an epidemic, will likely provide the biggest impact^35^. Until such network information can be made available in real time for each community, we feel that this tool can provide good targets for TasP and PrEP coverage rate in local populations to reduce incidence. This tool also does not account for any migration of HIV-infected or susceptible individuals. Depending on the rates and directions of such population movement, this could have a negligible or large effect on HIV incidence. In San Diego, this might be especially pertinent given the large bidirectional movement of people and HIV infections between San Diego, USA and Tijuana, Mexico^35^. Additionally, the model presented here does not allow for the prevalence of concurrent partnerships (i.e. partnerships that overlap in time)^36^, which may a differential impact on epidemic growth patterns among key populations^37^. Another limitation of the model is that it does not account for changes in the lifespan of an infected individual who does or does not receive ART. In general, the more ART uptake, the longer is the lifespan of infected individuals. In fact, a person living with HIV who has access to and adheres to ART can expect a relatively normal lifespan^38^,^39^. Such changes in lifespan in those on ART could have a large impact on total costs. In addition, we expect that costs of TasP and PrEP fluctuate over time (e.g. manufacturer costs, third party payor negotiations, inflation, generic pricing). Therefore, we provide only gross cost estimates per year of the TasP and PrEP interventions (i.e. price per set interval) and have allowed these parameters to adjusted within the model by the user. More complex cost modeling would be needed to infer the total costs for changes in the lifespan by providing ART to infected individuals and preventing infections among susceptible individuals. Finally, more detailed and more accurate cost analyses of PrEP and TasP coverage rates, sexual risk compensation and effective measure/mathematical measure of concurrency^40^ will be needed in order to determine cost-effectiveness of the scenarios presented here.

In summary, we currently have the tools needed to eliminate local HIV epidemics with TasP and PrEP. We do not, however, have benchmarks of coverage rates needed for this endeavor. With a limited number of parameters and epidemiologic estimates, we provide here an easy-to-use and flexible tool to estimate the impact of TasP and PrEP coverage rates. Such a tool should assist public health officials and policy makers in their decisions about how to focus resources.

*This tool is available online:* http://gtzero.ucsd.edu/.

## Additional Information

**How to cite this article**: Chaillon, A. *et al*. A practical online tool to estimate antiretroviral coverage for HIV infected and susceptible populations needed to reduce local HIV epidemics. *Sci. Rep.* **6**, 28707; doi: 10.1038/srep28707 (2016).

## Supplementary Material

Supplementary Information

## Figures and Tables

**Table 1:** Variables used to evaluate the San Diego HIV epidemic among men who have sex with men (MSM).

Variable	Definition	Estimates for MSM in San Diego	Ref.
HIV epidemic Estimates			
β	Risk of HIV transmission per sexual act =[(ß_RAI_*proportion of RAI) + (ß_IAI_*proportion of IAI)] *(1-μ) where:-ß (CRAI) = condomless receptive anal intercourse-ß (CIAI) = condomless insertive anal intercourse-μ = Frequency of condom use-*C* = number of sexual exposure per year	0.01380.001160%*10, 20 and 30*	14,14,12
*P*_*0*_	Initial total population size of MSM	56,000	
*π*	HIV prevalence	20%*	19
*P*_*(susceptible)*_	Initial population size of HIV exposed individuals	52,920	
*∂*	PrEP coverage rate	Negligible*	
*ƒ*	TasP coverage rate	30%*	
Efficacy of TasP	Percentage of individuals on ART who achieved viral suppression (TasP)	80%	13
Efficacy of PrEP	Percentage of individuals on PrEP who were considered protected	70%	3
Cost Estimates			
TasP	Average annual cost of HIV care	24,000USD/year	16
PrEP	Average annual cost of PrEP (FTC/TDF)#	10,300 USD/year	17

ART: Antiretroviral Therapy; PrEP: Pre Exposure Prophylaxis; TasP: Treatment as Prevention; FTC/TDF: combination oral emtricitabine/tenofovir disoproxil fumarate. *Adjustable variables; ^#^Annual cost of PrEP based on CDC recommendations for care, including drug costs, physician visits and laboratory testing^17^.
