# Supplementary material for: A practical online tool to estimate antiretroviral coverage for HIV infected and susceptible populations needed to reduce local HIV epidemics
Source: Sci Rep. 2016 Jun 24;6:28707. doi: 10.1038/srep28707 (PMC4919622; doi:10.1038/srep28707)

SUPPLEMENTARY MATERIAL

A practical online tool to estimate antiretroviral coverage for HIV infected and susceptible populations needed to reduce local HIV epidemics

Author's list:

Antoine Chaillon[1#], Martin Hoenigl[1,2,3], Sanjay R. Mehta[1,4], Nadir Weibel[5], Susan J. Little[1] and Davey M. Smith[1,4]

Affiliations:

[1] Division of Infectious Diseases, University of California, San Diego, La Jolla, California, United States of America

[2] Section of Infectious Diseases and Tropical Medicine, Department of Internal Medicine, Medical University of Graz, Graz, Austria

[3] Division of Pulmonology, Department of Internal Medicine, Medical University of Graz, Graz, Austria

[4] Veterans Affairs San Diego Healthcare System, San Diego, California, United States of America

[5] Department of Computer Science and Engineering, University of California San Diego, California, United States

#Corresponding Author:

Antoine Chaillon, MD

Division of Infectious Diseases,

UCSD & VA San Diego Healthcare System

UC San Diego

Stein Clinical Research Building #325 (mail code 0679)

9500 Gilman Drive, La Jolla, CA 92093

Telephone: (858) 552-7439

Email: achaillon@ucsd.edu

Running Title: Estimating TasP and PrEP coverage rates to reduce local epidemics

Supplementary Tables and Figures Legend.

Table S1. Potential impact of combined TasP and PrEP coverage on HIV incidence in San Diego. A. Number of new infections (NNI) after 1 year. B. Number of new infections (NNI) after 5 years. The number of new HIV infections (NNI) is indicated for each combined scenario of TasP and PrEP intervention. TasP coverage ranged from 0% to 60% (incremented by 10%) and PrEP coverage ranged from 10 to 50% (incremented by 10%); All estimates were made considering an initial population of 56,000, an average of 20 sex acts with casual partners per year, and an average condom use of 60%.

Table S2. Cumulative costs of TasP and PrEP among MSM in San Diego after one year (A)and after 5 years (B). The cumulative cost in million USD is indicated for each combined scenario of TasP and PrEP intervention. TasP coverage ranged from 30 to 60% (incremented by 10%) and PrEP coverage ranged from 10 to 50% (incremented by 10%). All estimates were made considering an initial population 56,000, an average of 20 sex acts with casual partners per year, and an average condom use of 60%.

Table S3. Impact of targeted PrEP based on age among MSM in San Diego (A) and cumulative cost after 5 years (B). A. Number of new HIV infections after 5 years. B. Cumulative cost after 5 years (in million USD).

Figure S1. Reported condom use among MSM in San Diego. Men presenting for testing who reported sex with other men were asked for the frequency of condom use during their receptive and insertive sex acts in four categorical answers: “never”, “sometimes” “mostly”, and “always”

Figure S2. Age distribution of HIV incidence (red) and male individuals (grey) in San Diego. Vertical dashed lines indicate the minimum (21 years) and maximum (52 years) of the MSM population associated with 90% of the new HIV infections among MSM in San Diego.

Figure S3. Cost estimates of TasP (A) and PrEP (B) coverage among MSM in San Diego. We considered a yearly cost of TasP and PrEP: 24,000$ and 10,300$ respectively and an average number of sexual acts with causal partner of 20/year. The cumulative cost estimates of TasP and PrEP are expressed in million USD$. Three different levels of TasP coverage (30%, 40% and 50%) and PrEP coverage (20%, 30% and 50%) and colored from light green to dark green. This analysis was based on an initial population size of 56,000 MSM individuals, an HIV prevalence of 20% among MSM, a mean number of sex acts of 10, 20 and 30/year, and 60% condom use.

Figure S4. Change in the annual cost of combined TasP and PrEP coverage (A) and estimated number of new HIV infections averted among MSM in San Diego (B). Results are indicated for an average annual number of sex acts with causal partner of 20/year and for PrEP coverage levels of 0%, 30% and 50% respectively. TasP coverage levels are indicated in the circles (30, 40 and 50%)* the annual cost of TasP would be estimated to decrease after 10 years

Figure S5. Cost estimates by targeting PrEP to MSM between the ages of 21 and 52 years. Costs in million USD are estimated for a PrEP coverage of 20% with an average number of sex acts with causal partner is 20/year.

Table S1. Potential impact of combined TasP and PrEP coverage on HIV incidence in San Diego.

A. Number of new infections (NNI) after 1 year.

TasP Coverage	PrEP Coverage: 0%	10%	20%	30%	40%	50%
**0%**	638	593	549	504	459	415
**30%**	449	418	386	355	323	292
**40%**	383	356	329	302	276	249
**50%**	316	294	272	250	228	206
**60%**	255	237	219	202	184	166

B. Number of new infections (NNI) after 5 years.

TasP Coverage	PrEP Coverage: 0%	10%	20%	30%	40%	50%
**0%**	3282	3052	2822	2593	2363	2133
**30%**	2291	2131	1970	1810	1650	1489
**40%**	1947	1811	1674	1538	1402	1265
**50%**	1605	1492	1380	1268	1155	1043
**60%**	1291	1200	1110	1020	929	839

The number of new HIV infections (NNI) is indicated for each combined scenario of TasP and PrEP intervention. TasP coverage ranged from 0% to 60% (incremented by 10%) and PrEP coverage ranged from 10 to 50% (incremented by 10%); All estimates were made considering an initial population of 56,000, an average of 20 sex acts with casual partners per year, and an average condom use of 60%.

Table S2. Cumulative costs of TasP and PrEP among MSM in San Diego.

A. After one year.

TasP Coverage	PrEP Coverage 0%	10%	20%	30%	40%	50%
**0%**	0	40	79	120	159	199
**30%**	99	138	177	217	258	292
**40%**	132	171	210	249	288	327
**50%**	163	202	241	280	320	358
**60%**	191	230	269	308	347	387

B. After 5 years.

TasP Coverage	PrEP Coverage 0%	10%	20%	30%	40%	50%
**0%**	0	193	388	584	781	979
**30%**	535	718	902	1088	1275	1462
**40%**	687	870	1053	1238	1422	1608
**50%**	821	1004	1188	1373	1558	1743
**60%**	930	1114	1299	1485	1670	1857

The cumulative cost in million USD is indicated for each combined scenario of TasP and PrEP intervention. TasP coverage ranged from 30 to 60% (incremented by 10%) and PrEP coverage ranged from 10 to 50% (incremented by 10%). All estimates were made considering an initial population 56,000, an average of 20 sex acts with casual partners per year, and an average condom use of 60%.

Table S3. Impact of targeted PrEP based on age among MSM in San Diego (A) and cumulative cost after 5 years (B).

A. Number of new HIV infections after 5 years.

		PrEP Coverage targeted to high-risk MSM by age#					
		**0%**	**10%**	**20%**	**30%**	**40%**	**50%**
**TasP Coverage**	**0%**	3282	3075	2868	2662	2455	2248
	**30%**	2291	2147	2002	1858	1714	1569
	**40%**	1947	1824	1702	1579	1456	1334
	**50%**	1605	1504	1402	1301	1200	1099
	**60%**	1291	1209	1128	1047	965	884

The number of new HIV infections (NNI) is indicated for combined coverage of TasP and targeted PrEP.

B. Cumulative cost after 5 years (in million USD).

		PrEP Coverage targeted to high-risk MSM by age#					
		**0%**	**10%**	**20%**	**30%**	**40%**	**50%**
**TasP Coverage**	**0%**	0	90	180	270	362	453
	**30%**	535	615	696	778	860	992
	**40%**	687	773	853	933	1014	1095
	**50%**	821	917	997	1077	1157	1238
	**60%**	930	1038	1119	1199	1280	1362

All costs are indicated in Million USD; All estimates were made considering an initial population of 56,000, an average of 20 sex acts with casual partners per year, and an average condom use of 60%. #PrEP targeted on MSM between the ages of 21 and 52 years.

Figure S1. Figure S1. Reported condom use among MSM in San Diego.

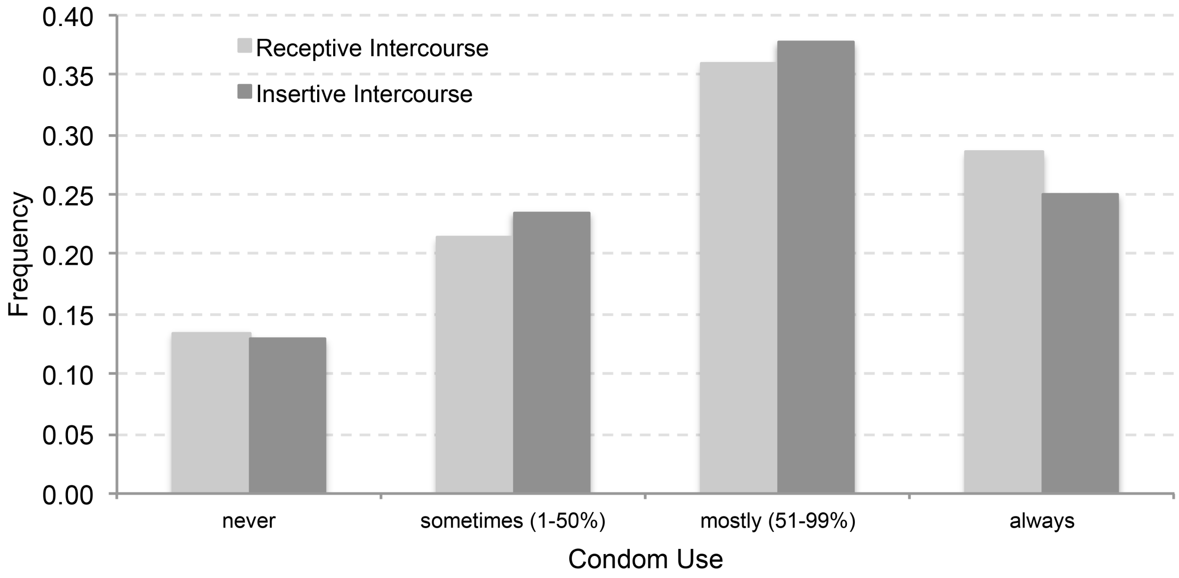

Figure S2. Age distribution of HIV incidence (red) and male individuals (grey) in San Diego.

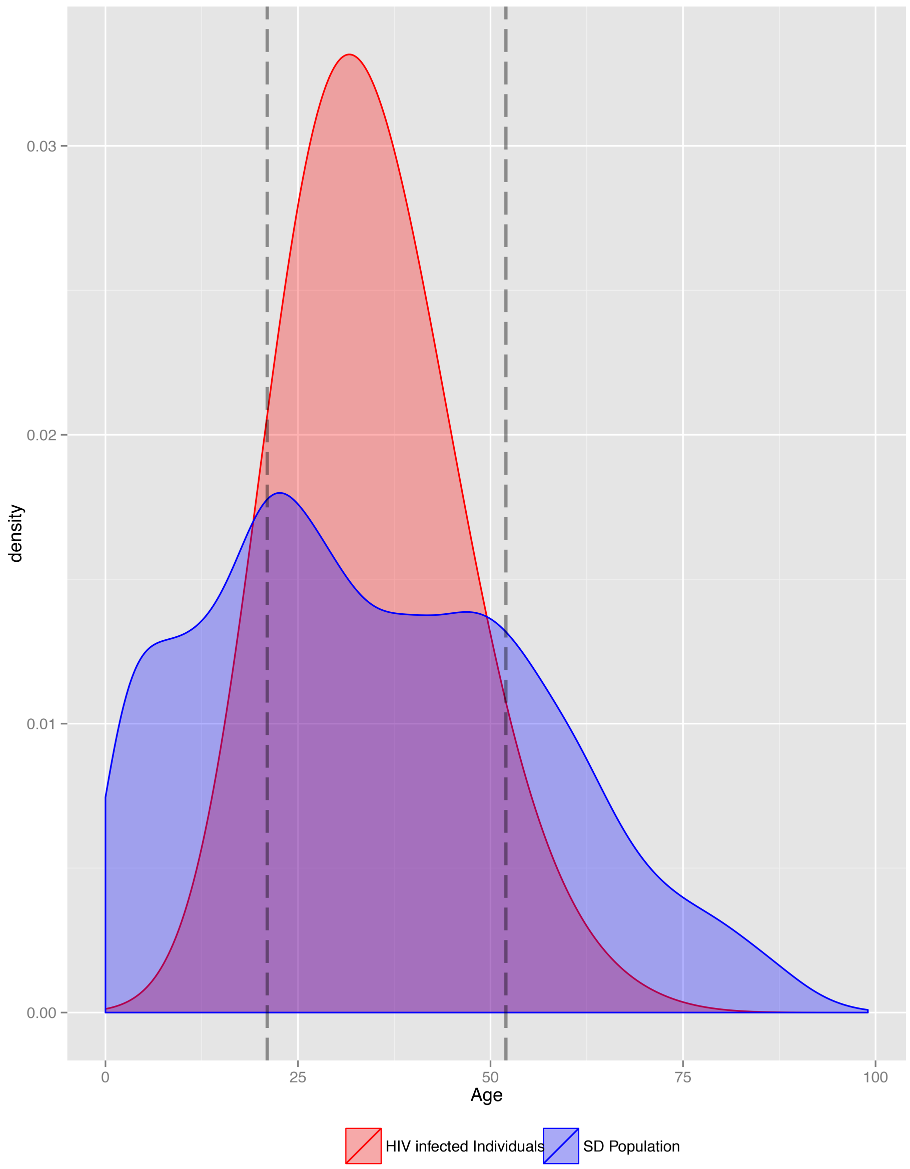

Figure S3. Cost estimates of TasP (A) and PrEP (B) coverage among MSM in San Diego.

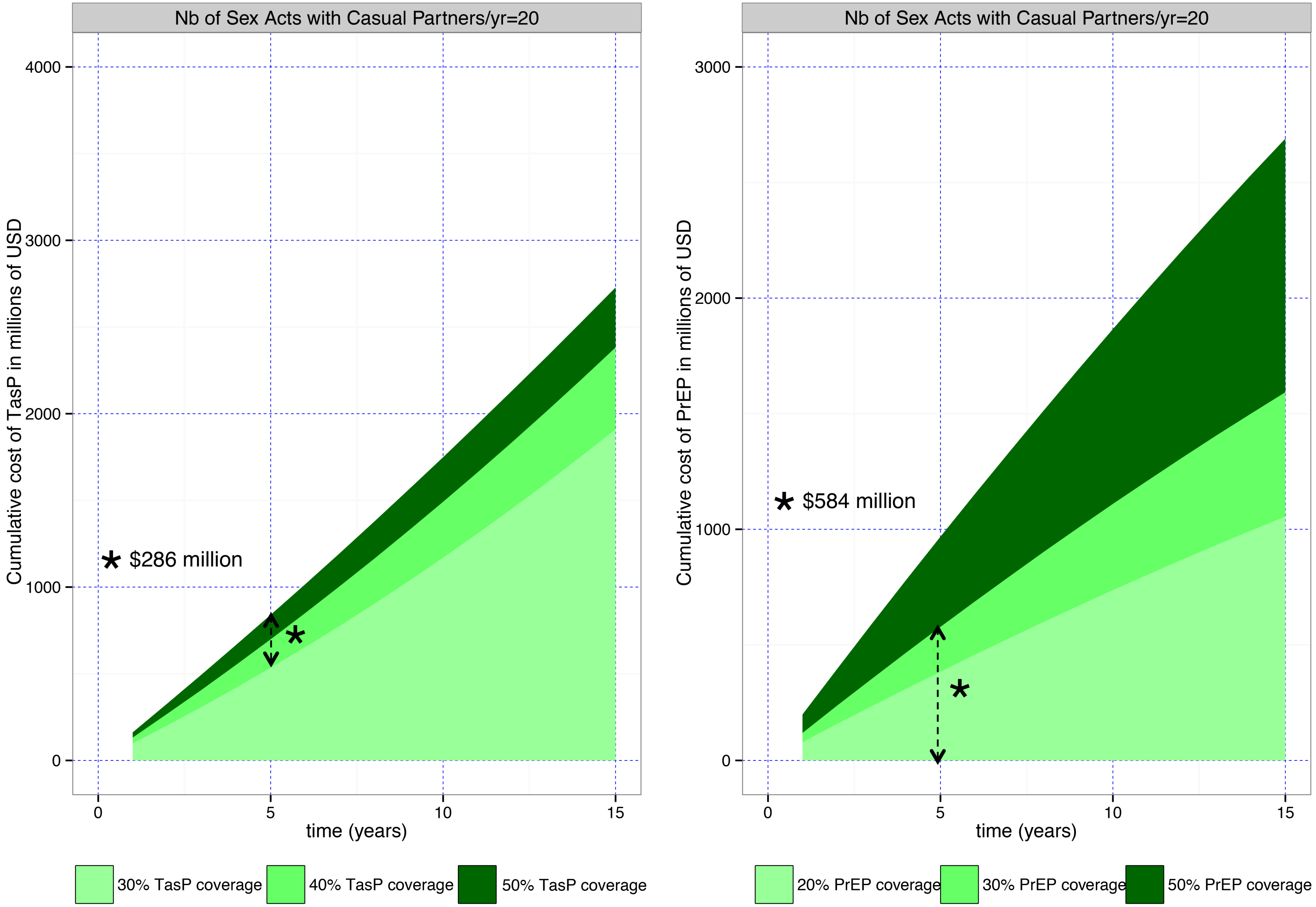

Figure S4. Change in the annual cost of combined TasP and PrEP coverage (A) and estimated number of new HIV infections averted among MSM in San Diego (B).

A.

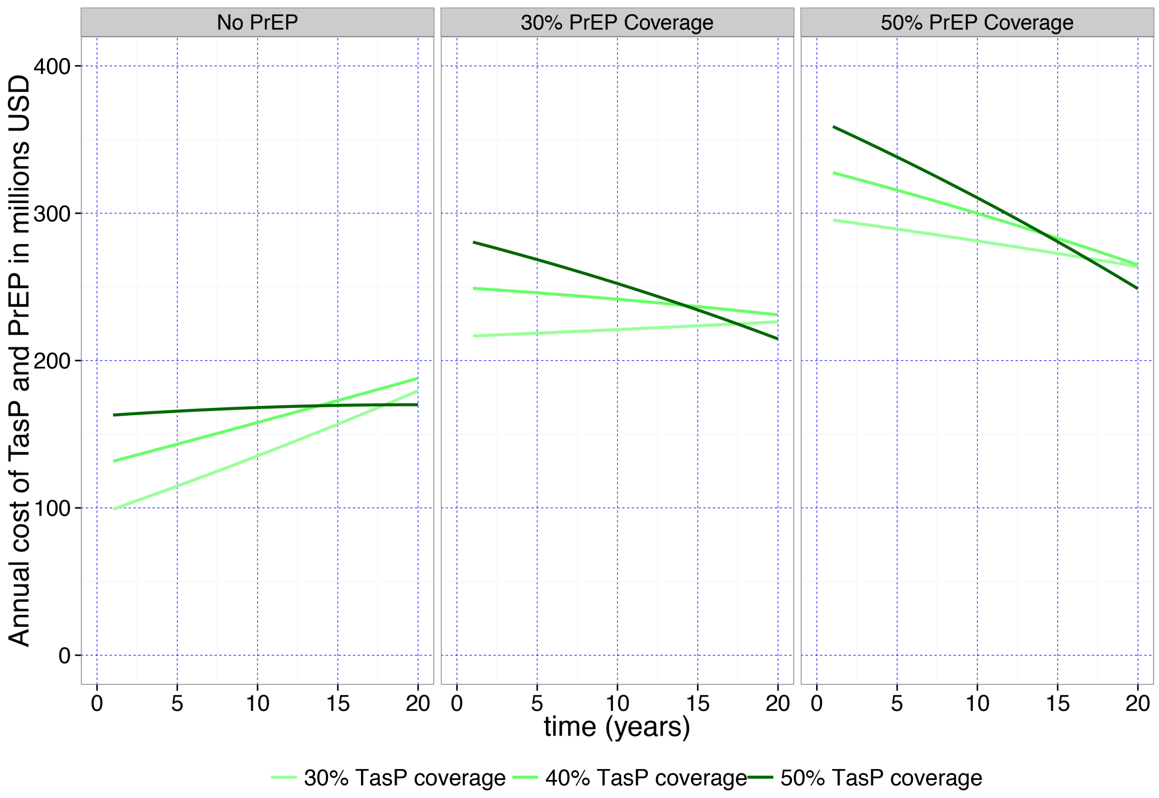

B.

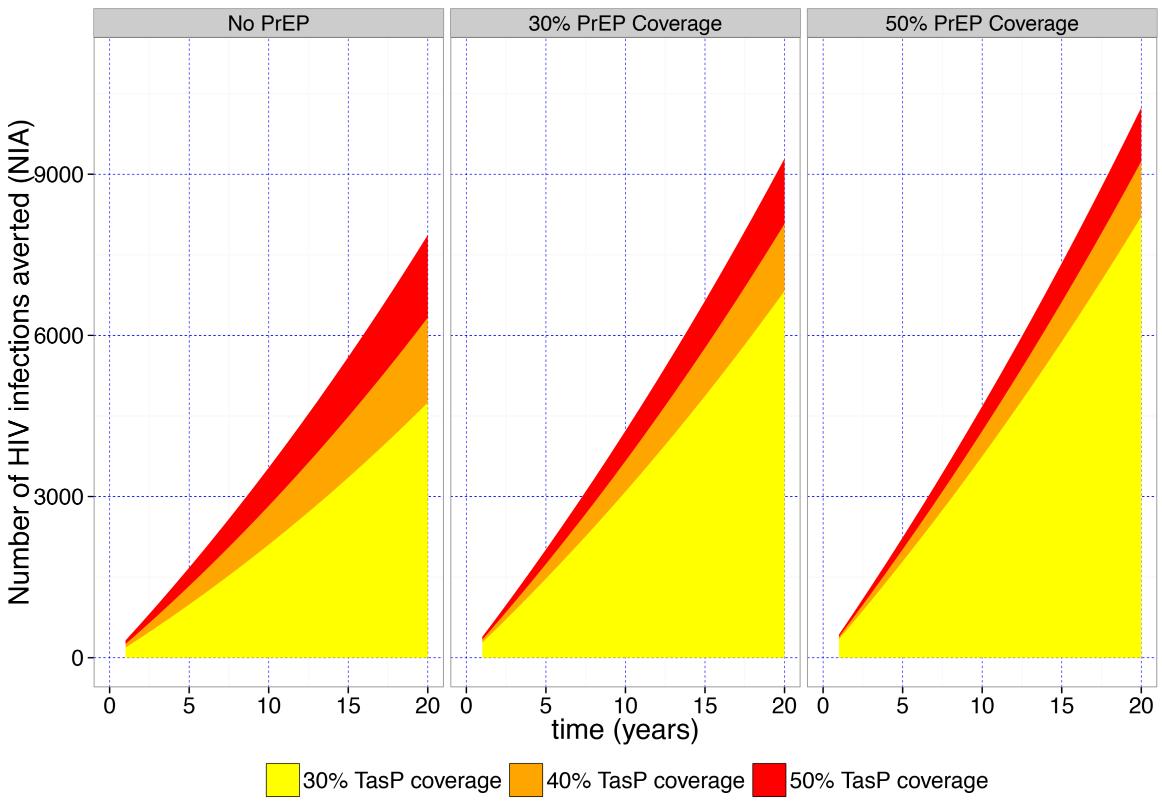

Figure S5. Cost estimates by targeting PrEP to MSM between the ages of 21 and 52 years.

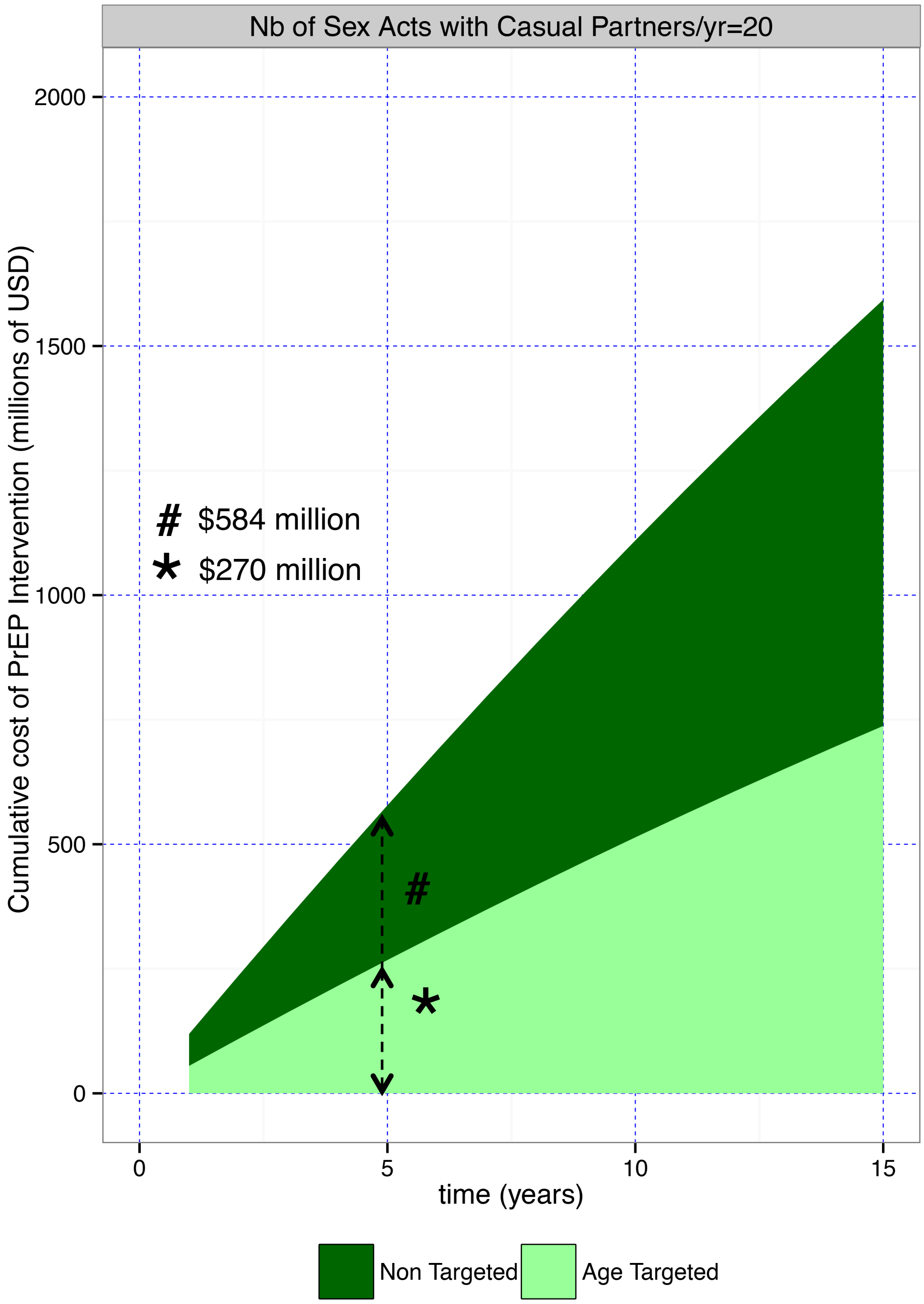

Supplement: Supplementary Information [file srep28707-s1.pdf]
